# The use of small diameter nozzles in temperature-controlled hemp oil extraction allows high oil yields and good quality residual hemp cake feed

**DOI:** 10.3389/fvets.2023.1322637

**Published:** 2024-01-05

**Authors:** Maura Sannino, Alessandro Vastolo, Salvatore Faugno, Felicia Masucci, Antonio Di Francia, Fiorella Sarubbi, Maria Eleonora Pelosi, Dieu donnè Kiatti, Francesco Serrapica

**Affiliations:** ^1^Department of Agriculture, University of Naples Federico II, Portici, Italy; ^2^Department of Veterinary Medicine and Animal Production, University of Naples Federico II, Napoli, Italy; ^3^Institute for the Animal Production System in the Mediterranean Environment, National Research Council, Portici, Italy

**Keywords:** hemp seeds, oil extraction parameters, nozzle size, oil press cake composition, *in vitro* fermentation

## Abstract

The use of two nozzle diameters (6 and 8 mm) in a cold (50°C) hemp seed oil extraction process was evaluated in terms of extraction efficiency, and chemical composition and *in vitro* fermentation characteristics of the residual cake. Seeds of the varieties Futura 75 and Uso 31 were pressed using a mechanical press with a cooling device. Five pressings were carried out for each variety and nozzle size, the functional parameters of the extraction processes were recorded, and sample of the residual cakes (*n* = 20) were analyzed. The 6 mm nozzle determined a higher oil yield (+4%) with a limited increase in temperature in the pressing chamber and in the oil (on average + 3°C compared to the 8 mm nozzle). A lower oil yield and consequently a higher fat content in the corresponding cake was observed when using the 8 mm nozzle. Despite the similar fat content, the two varieties had different oil yields and different residual cake compositions. The gas production kinetic of cakes was influenced by variety but little by nozzle size. Overall, the use of a smaller nozzle in a temperature-controlled extraction process can be a useful option to increase hemp oil yield while maintaining good fermentation characteristics of the residual cakes as ruminant feed.

## Graphical Abstract

Created with BioRender.com.

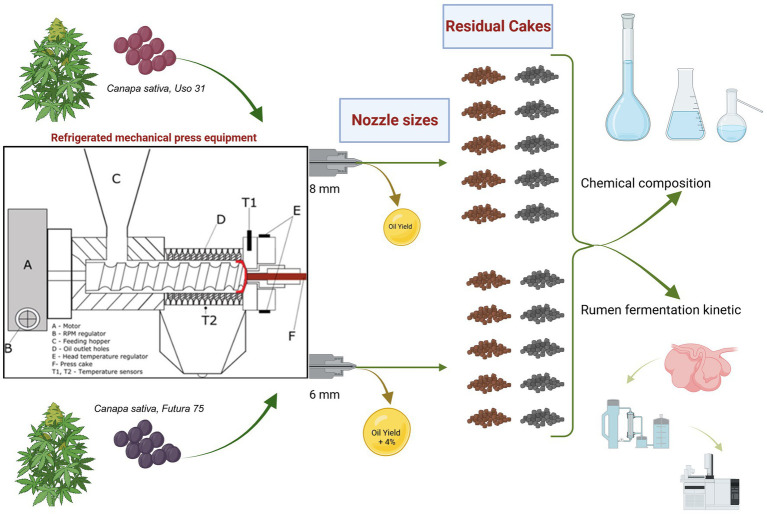

## 1 Introduction

Hemp is a low-cost, environmentally friendly, and versatile crop that can provide fiber for industrial applications as well as oilseeds and derived products for human and animal consumption (1, 2). Unlike more widespread oilseeds such as soybean and rapeseed, hemp can be easily cultivated in Mediterranean areas with low rainfall and the seeds are suitable for processing in small and medium extraction plants (3, 4). In addition to its use in the food industry, the hemp cake left over from mechanical oil extraction is widely used as an animal feed (2, 5) due to its medium/high protein content of high bio-logical value with an amino acid profile comparable to that of soybean (6). In addition, hempseed cake can be used as a feed supplement to provide essential fatty acids and essential amino acids (7). For this reason, hemp has been proposed as a crop that can both diversify the income of small farms in the form of oilseed and provide a source of locally produced protein for livestock in the form of cakes (8). However, the high compositional variability of hempseed cake, which may be due to both the quality of the unprocessed seeds and the oil extraction process, may limit its use as animal feed (3). Hemp seeds generally contain around 33–35% oil, which is mainly composed of ω3 and ω6 essential fatty acids (9). However, the high content of polyunsaturated fatty acids (PUFA) makes hemp oil susceptible to oxidative processes, so careful management of seed harvesting and storage, as well as the oil extraction process, is necessary to ensure a high-quality oil (10). Separation of the oil phase from the solid phase of oilseeds by mechanical extraction using a screw press is the oldest extraction method still used to produce fine oils (11). Indeed, mechanical extraction is widely used in small and medium-sized extraction plants due to its advantages in terms of plant operating costs, oil safety and the environmental impact of the process due to the absence of solvents (12). On the other hand, compared to solvent extraction, mechanical extraction is less efficient in terms of oil yield, so modulation of temperature and pressure parameters, along with process strategies such as seed preheating and screw rotation speed, have been studied to increase oil yield (9). However, increased temperature can affect the fatty acid composition of the oil due to the thermal instability of PUFAs (13, 14). Nozzle size is another critical factor in seed oil extraction, as it affects the pressure in the pressing chamber and thus the oil yield and quality, as well as the efficiency of the extraction process. In particular, reducing nozzle size increases extraction pressure and thus oil yield, but can also reduce extraction efficiency in terms of kg of oil per hour and, most importantly, increases extraction temperatures, which in turn negatively affects the chemical and nutraceutical properties of the oil (15, 16).

Sporadic reports have investigated the effect of nozzle size on hemp oil yield and quality (10, 17–19), but none of them addressed the issue of extraction temperature and of the residual cake quality. Therefore, this study analyzed the use of two nozzle diameters in a cold extraction process (50°C) (i) on the pressing efficiency (ii) on the chemical composition and *in vitro* fermentation characteristics of hemp cake. The oil properties will be the subject of a further report.

## 2 Materials and methods

### 2.1 Seed production, mechanical extraction and sampling

**Figure 1:**
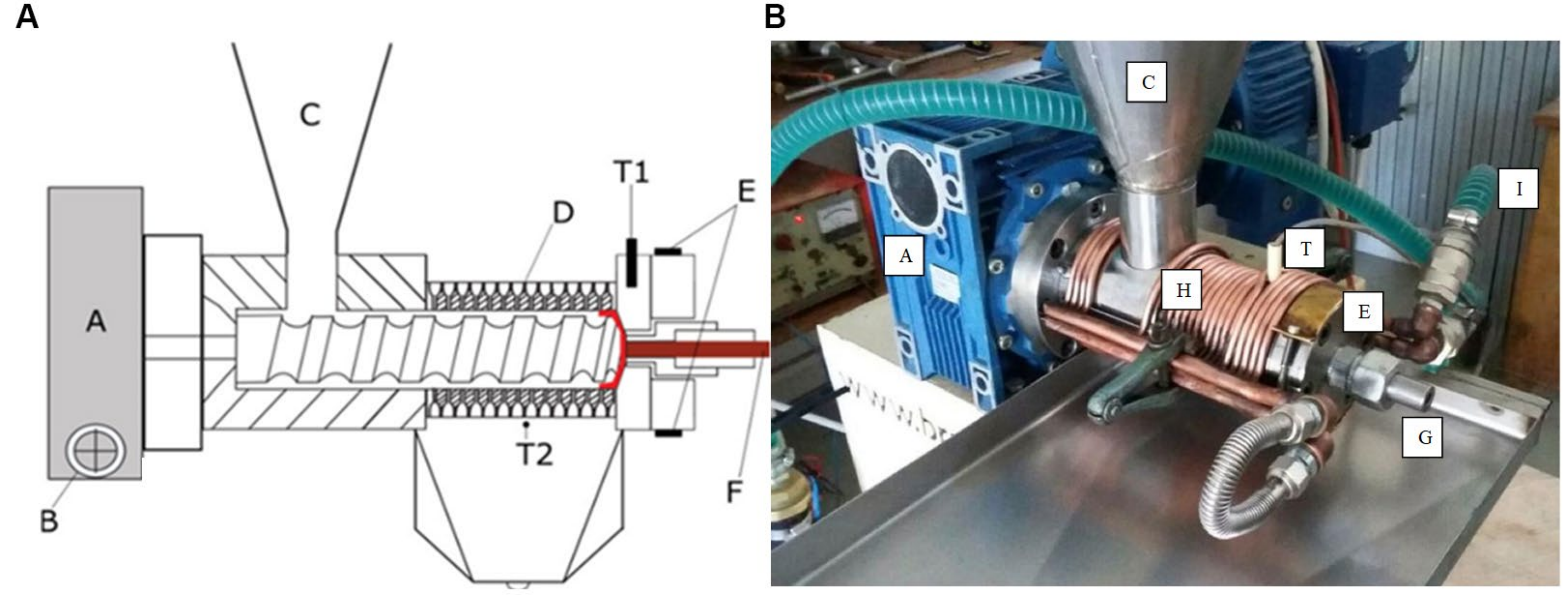
Mechanical screw press for hemp oil extraction. **(A)** Mechanical components and temperature sensors [adapted from Faugno et al. (22)]. **(B)** Cooling system. A – Motor; B – RPM regulator; C – Feeding hopper; D – Oil outlet holes; E- Head temperature regulator; F – Pressed cake; G- Nozzle; H – Copper oil; I – Coolant Fluid; T – Temperature sensor.

The hemp varieties tested were Futura 75 and Uso 31, late and early flowering respectively, both registered in the EU Common Catalog and commercially available (Δ9-THC ≤ 0.2%). The varieties were sown with a standard seed drill in April 2021 in lowland fields (40°55′N 14°15′E; 27 m a.s.l.) with loose sandy soils. The sowing rate and depth were 50 kg/ha and 2–3 cm respectively, which are the recommended values for hemp seed production (20). The crops were not irrigated and were harvested by threshing in July, giving average yields of 1.2 and 1.3 t/ha seed for Futura 75 and Uso 31, respectively. The seeds were transported to an oil extraction plant, where they were cleaned with a pneumatic cleaner to remove impurities (21, 22), dried with an air stream heated to 40°C in a cross-flow dryer equipped with a screw to ensure an even distribution of the air stream over the seeds, and sorted with a sieving machine consisting of four oscillating sieves that removed hollow seeds, coarse dust and small stones. The seeds were finally stored in sealed jars at a temperature between 15 and 25°C, as the moisture content (8%), determined in an air oven (BD115, BINDER GmbH, Tuttlingen, Germany) set at 105 ± 1°C, was low enough to prevent microbial spoilage (23). Seeds of each variety were pressed for oil using a Bracco screw press (Bracco s.r.l., Bagnatica, Bergamo, Italy) powered by a 2.2 kW electric motor and equipped with a heat exchanger to maintain constant the extraction temperature into the press chamber as well as sensors to measure temperature and screw rotation speed (21) (Figures 1A,B).

The cooling system (Figure 1B) consisted of a copper coil wrapped outside the com-pression chamber, in which a coolant (food grade propylene glycol diluted 40% with dis-tilled water) circulated. The coil was connected to a liquid chiller and pump to provide a continuous flow of cold coolant in the circuit. The length of the coil, the temperature of the coolant and the speed of the pump were set to achieve an extraction temperature of 50°C within the compression chamber, regardless of nozzle diameter. The temperature in the compression zone (T1), where the highest operating values were expected, was measured using a resistance temperature detector (RTD), such as the Pt100. Another sensor measured the temperature of the oil extracted (T2) immediately after it exited the holes in the press cylinder. The speed of the screw was controlled by a variable speed controller and measured by a tachometer. To record the sensor data, a compact-DAQ chassis (Model cDAQ-9171, National Instruments, Austin, TX, United States) was employed, and the data were transferred to a laboratory computer using LabVIEW 2014 software. The mechanical press was equipped with four nozzles of different sizes (6; 8; 10; 12 mm). Five preliminary pressing trials were carried out, and in relation to the size of the hemp seeds, the 6 and 8 mm nozzles gave the best results (21). During the extraction process, a constant extraction temperature of 50°C and a screw speed of 22 rpm were set (22). The throughput of the cold press (TP, in kg/h) was calculated as the operating capacity by dividing the weight of hemp seeds (S, in kg) by the time (t, in h) required to complete the pressing process. The energy consumption (ES, in kWh kg^-1^ of processed seeds) was calculated by dividing the total energy (Et, in kWh) by the sample size (S, in kg). To calculate Et, the average power (P, in kW) was multiplied by the average pressing time (t, in h) required for each sample (21). Five extractions were performed for each diameter and variety using 1,000 g of seed weighed on a technical scale with an accuracy of ±0. 1 g (24, 25). For each extraction, preparatory steps were conducted, including press cleaning, setting the press head temperature using an electrical resistance (21), and adjusting the screw speed. Oil was collected in lightproof glass containers. To remove settled components from storage, the crude oil was subjected to centrifugation using an ALC centrifuge (ALC T535 PK130R, Winchester, United States) at 3500 rpm for 20 min. Raw and purified oil yield was assessed. The remaining hempseed cakes after oil extraction (n. 20, i.e., 2 varieties x 2 nozzles x 5 replicates) and unpressed seed samples were stored in vacuum packs at 4°C in the dark until analysis, which was performed within 3 weeks.

### 2.2 Chemical analysis

The seed and cake samples were ground at a laboratory mill (Brabender Wiley mill, Brabender OHG Duisburg, Germany) equipped with a 1.1 mm grid to be analyzed according to Association of Official Analytical Chemists (26) for dry matter (DM; method 930.15), ash (method 942.05), crude protein (CP; N x 6.25, method 976.05), and ether extract (EE; method 954.02) and according to Van Soest (27) and Robertson and Van Soest (28) for neutral detergent fiber (NDF), acid detergent fiber (ADF), inclusive of residual ash, and acid detergent lignin (ADL).

### 2.3 *In vitro* gas production

The cake samples were incubated in a serum flask (seven replications per substrate ±1 g for each replication) with buffalo rumen fluid (10 mL) at 39°C under anaerobic conditions as indicated by Theodorou et al. (29). The rumen liquor was collected at a slaughterhouse from four buffaloes according to the procedure described by Serrapica et al. (3). The buffaloes fed a total mixed ration containing corn silage, oat hay and concentrate. All procedures involving animals were approved by the Ethical Animal Care and Use Committee of the University of Napoli Federico II (Prot. 2019/0013729 of 08/02/2019). The collected rumen fluids were placed inside to preheated thermos and transported within 2 h to the laboratory of Department of Veterinary and Animal Production, University of Napoli Federico II. The rumen fluid was mixed and strained through four layers of cheese cloths and diluted in a buffered medium (75 mL); successively, the reducing agent (4 mL) was added into the flasks (30, 31). On seven replication, four bottles for each substrate were utilized for cumulative gas production measurement (48 h of incubation). The gas produced during 48 h of incubation, into the fermenting cultures, was recorded 10 times (from 2 to 24 h of intervals) using a manual pressure transducer (Cole and Palmer Instrument Co, Vernon Hills, IL, United States). The cumulative volume of gas produced after 48 h of incubation was related to incubated organic matter (OMCV, ml/g). At the end of the incubation period, the fermentation liquor was analyzed for pH using a pH meter (ThermoOrion 720 A+, Fort Collins, CO, United States). The organic matter degradability (OMD, %) was determined by weight difference of the incubated organic matter (OM) and the undegraded filtered (sintered glass crucibles; Schott Duran, Mainz, Germany, porosity # 2) residue burned at 550°C for 3 h.

### 2.4 End-products analysis

To determine the volatile fatty acids (VFA), the fermentation liquor was cooled at 4°C and, before analyzes, centrifuged at 12,000 g for 10 min at 4°C (Universal 32R centrifuge, Hettich FurnTech Division DIY, Melle- Neuenkirchen, Germany); the supernatant (1 mL) was then mixed with 1 mL of 0.06 mol oxalic acid. The VFA was measured by gas chromatography (ThermoQuest 8000top Italia SpA, Rodano, Milan, Italy) equipped with a fused silica capillary column (30 m, 0.25 mm ID, 0.25 μm film thickness), using an external standard solution composed of acetic, propionic, butyric, iso- butyric, valeric and iso- valeric acids. The percentage of branched-chain fatty acids (BCFA) were calculated as: (iso-butyric acid +iso-valeric acid/VFA)/100.

### 2.5 Data processing

For each bottle stopped at 48 h, the gas production profiles were fitted to the sigmoidal model to estimate the fermentation kinetics (32):


$$G=A∕{\left(1+B∕t\right)}^{C}$$

where G is the total gas produced (ml per g of incubated OM) at time t (h), A is the asymptotic gas production (ml/g), B is the time at which one-half of A is reached (h), and C is the curve switch. Maximum fermentation rate (R_max_, ml/h) and the time at which it occurs (T_max_, h) were calculated utilizing model parameters (33):


$$R_{\text{max}}=\frac{\left(A\;x\;C^{B}\right)\;x\;B\;x\;{T_{\text{max}}}^{\left(B-1\right)}}{\left(1+C^{B}\right)x\;{\left(T_{\text{max}}-B\right)}^{2}}$$

$$T_{\text{max}}=C\;x\;{\left(\frac{B-1}{B+1}\right)}^{1/B}$$

### 2.6 Statistical analysis

Statistical analysis was performed by using JMP 14.0.0 software (SAS Institute Inc., Cary, NC, United States, 1989–2019). Data on seed chemical composition were analyzed by one-way ANOVA with hemp variety as the main effect. Functional parameters of the press, oil yield, and chemical composition, *in vitro* parameters and end-products of the cakes were analyzed by two-way ANOVA with hemp variety, nozzle diameter and interaction as main effects. Significance level was verified by t-student test at *p* < 0.05. Tendency was discussed at *p* < 0.10.

## 3 Results

Seed chemical composition is shown in Table 1. The differences between the two cultivars were limited to CP (*p* = 0.058) and ash content (*p* = 0.0013), with Futura 75 showing better quality (i.e., higher CP and lower ash).

**Table 1:** Chemical composition (% dry matter) of the hemp seeds (least square mean).

Item	Variety		SEM	Significance^1^
	Futura 75	Uso 31		
Dry matter %	92.0	91.9	0.30	NS
Ash	5.2	7.5	0.18	**
Crude protein	20.9	19.9	0.17	+
Ether extract	23.9	23.2	0.12	NS
NDF	48.8	48.6	0.51	NS
ADF	42.7	42.5	0.25	NS
ADL	13.2	13.4	0.21	NS

^1^**p ≤ 0.01; + indicates *p* ≤ 0.10; NS (not significant) indicates *p* > 0.10. SEM, standard error of means NDF, neutral detergent fiber; ADF; acid detergent fiber; ADL, acid detergent fiber.

Table 2 shows the functional parameters of the cold press as influenced by nozzle size (8 mm and 6 mm) and hemp varieties (Uso 31 and Futura 75).

**Table 2:** Selected functional parameters (least square mean) for the cold oil extraction.

Item	Nozzle diameter		Variety		SEM	Significance^1^		
	8 mm	6 mm	Futura 75	Uso 31		Diameter	Varieties	Interaction
T1, °C	51.04	54.07	52.52	52.59	0.06	***	NS	NS
T2, °C	21.22	24.30	22.80	22.72	0.51	***	NS	NS
t, min	4.33	4.38	4.34	4.37	0.04	NS	NS	NS
TP, kg/h	11.78	11.54	11.76	11.56	0.29	NS	NS	NS

^1^***p ≤ 0.001; NS (not significant) indicates *p* > 0.10.

T1, compression zone temperature; T2, extracted oil temperature; t, extraction time; TP, throughput. SEM, standard error of means.

The use of the 6 mm nozzle significantly (*p* < 0.001) increased the temperature of both the compression zone (on average, 3.0 ± 1.7°C compared to the T 50° initial temperature) and the extracted oil (on average 3.1 ± 1.3°C) but did not affect the throughput as operational capacity or the extraction time. No effects of hemp variety were observed for any of the functional parameters. The influence of nozzle diameter and hempseed variety on oil yield and residual cake weight and composition are shown in Table 3. The use of the 6 mm-nozzle resulted in higher oil yield and consequently lower cake weight (*p* < 0.001) compared to the larger 8 mm-nozzle. In addition, the 6 mm nozzle little but significantly reduced in oil dregs (*p* = 0.0085). The diameter of the nozzle size influenced also almost all chemical characteristics of residual cakes except CP, for which only a tendency (*p* = 0.09) to a slightly higher value was observed for the 8 mm cakes. In particular, the 6 mm nozzle showed a lower EE and ash content and an increment of the percentages of fiber fractions (i.e., NDF, ADF and ADL).

**Table 3:** Oil yield and cake chemical characteristics (least square mean) as affected by nozzle size diameter, varieties, and their interaction.

Item	Nozzle diameter		Variety		SEM	Significance^1^		
	8 mm	6 mm	Futura 75	Uso 31		Diameter	Variety	Interaction
Oil yield, g/kg seeds								
Cake weight	768.0	737.4	772.8	732.5	0.97	***	***	NS
Raw oil	232.0	262.6	227.2	267.4	0.97	***	***	NS
Oil	224. 6	255.3	219.8	260.0	0.96	***	***	NS
Oil-dregs	7.4	7.3	7.4	7.4	0.03	**	NS	NS
Cake chemical characteristics, % dry matter								
Dry Matter %	93.2	92.6	93.0	92.7	0.26	NS	NS	NS
Ash	7.1	6.9	6.4	7.6	0.04	*	***	NS
Crude Protein	23.6	23.2	23.8	22.9	0.14	+	**	NS
Ether Extract	8.8	7.8	8.7	7.9	0.16	**	*	NS
NDF	546	55.8	57.5	53.0	0.15	***	***	NS
ADF	42.5	43.5	43. 7	42.4	0.15	**	***	***
ADL	14.7	15.4	16.4	13.7	0.13	**	***	*

^1^*p ≤ 0.05; **p ≤ 0.01; ***p ≤ 0.001; + indicates *p* ≤ 0.10; NS (not significant) indicates *p* > 0.10. SEM, standard error of means; NDF, neutral detergent fiber; ADF; acid detergent fiber; ADL, acid detergent fiber.

As far as the influence of hemp variety was concerned, both the yield of oil and the chemical composition of the cakes were affected. The Uso 31 had a higher oil yield and consequently a lower cake weight then Futura 75. In addition, the Uso 31 cakes showed a lower protein (*p* < 0.0001) and fat (*p* < 0.01) contents and a higher ash (*p* < 0.0001) and fiber percentages (*p* < 0.0001).

The *in vitro* gas production parameters are shown in Table 4.

**Table 4:** *In vitro* gas production parameter of tested pellet (least square mean).

Item	Nozzle diameter		Variety		SEM	Significance^1^		
	8 mm	6 mm	Futura 75	Uso 31		Diameter	Variety	Interaction
OMD, %	29.8	29.1	27.1	31.8	6.64	NS	***	NS
OMCV, ml/g	93.3	92.9	96.1	93.1	7.11	*	*	NS
R_max_, ml/h	4.03	3.25	3.65	3.63	0.62	*	NS	NS
T_max_, h	5.17	6.55	6.14	5.58	2.83	NS	NS	NS

^1^*p ≤ 0.05; ***p ≤ 0.001; NS (not significant) indicates *p* > 0.10.SEM, standard error of means.

OMD, Organic Matter degradability; OMCV: cumulative volume of gas related to incubate organic matter; R_max_: maximum fermentation rate; T_max_: time at which R_max_ occurs. SEM, standard error of means.

**Figure 2:**
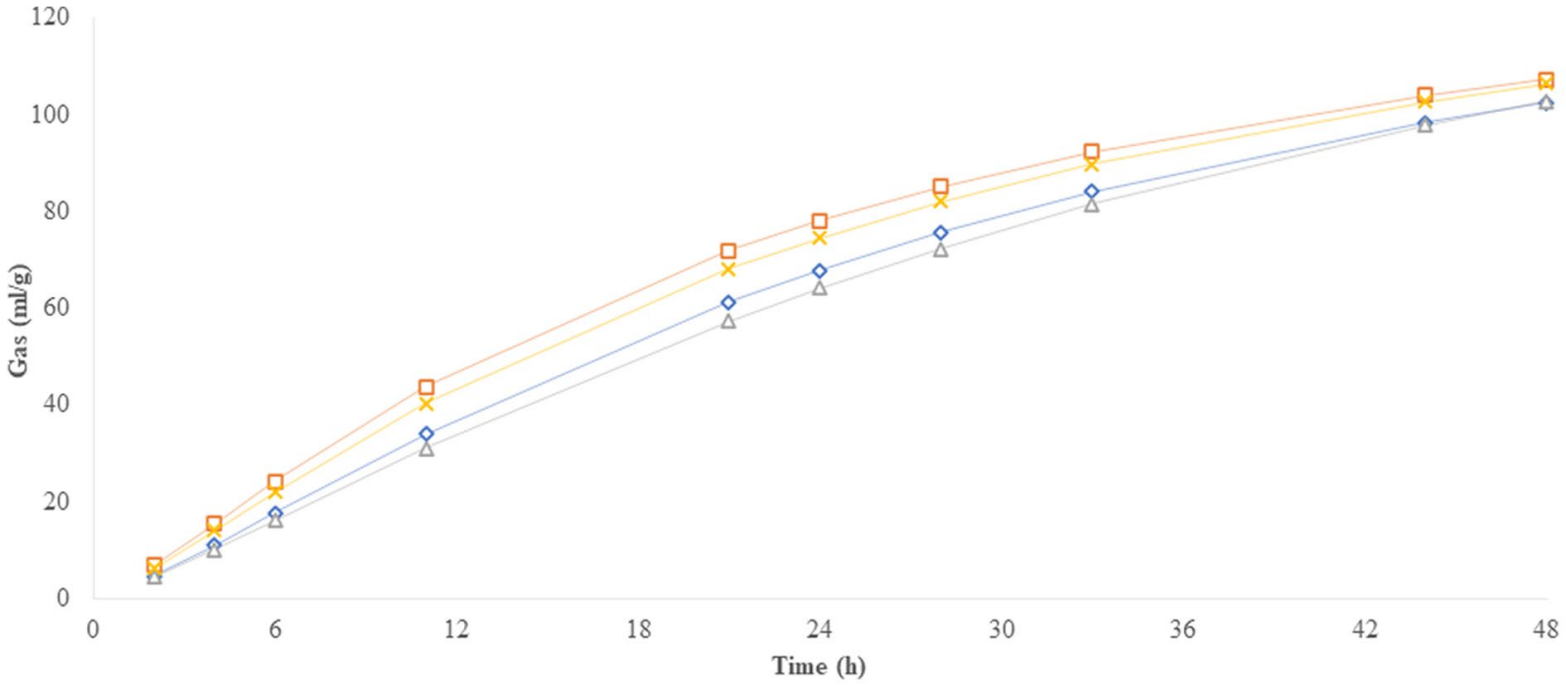
*In vitro* gas production over time.

**Figure 3:**
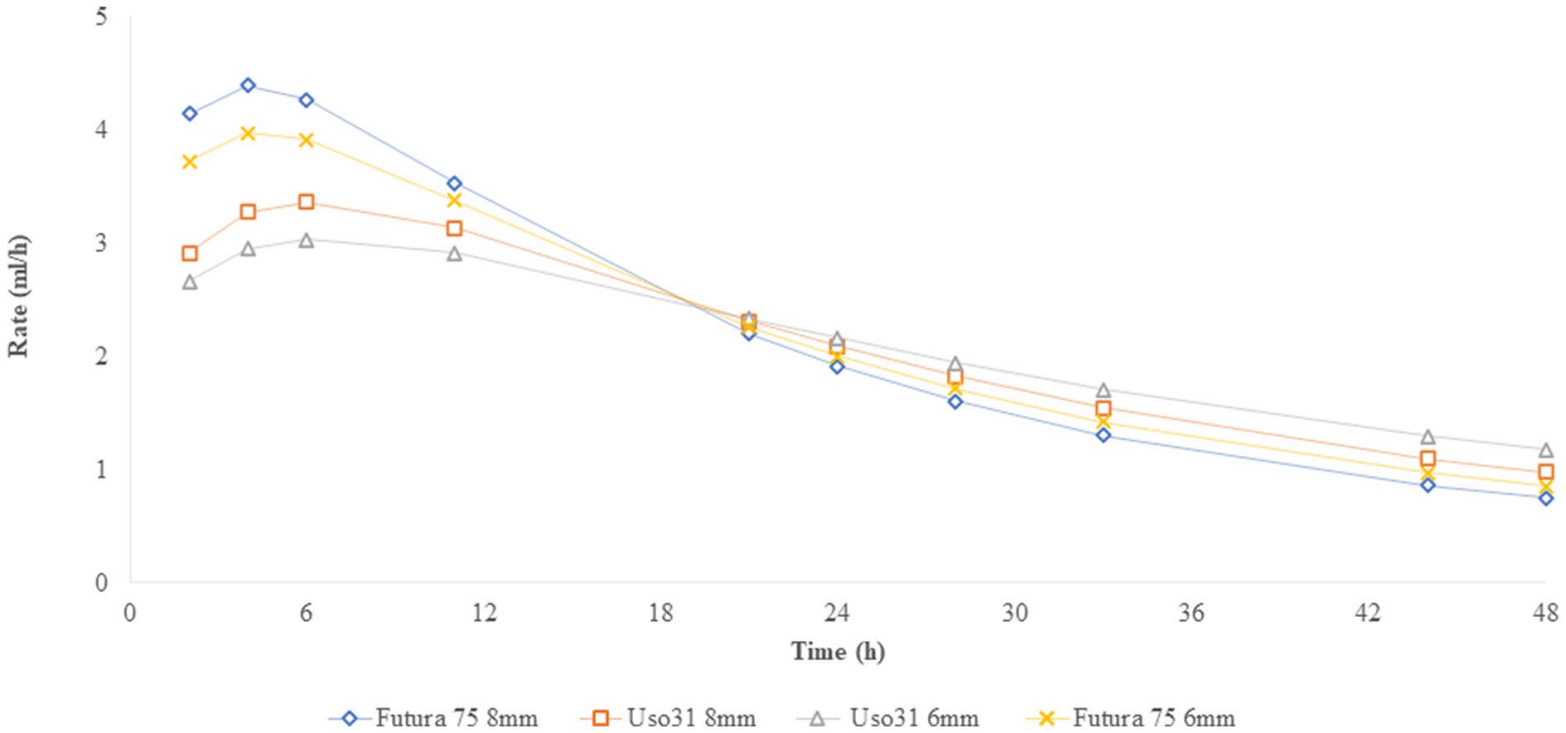
*In vitro* fermentation rate.

Compared to the 6 mm cakes, the 8 mm cakes produced a higher (*p* < 0.05) amount of gas (OMCV) and showed a faster (*p* < 0.05) fermentation rate (R_max_), while no differences were observed for organic matter degradability (OMD) and time to R_max_. The Uso 31 variety showed a higher OMD percentage (*p* < 0.01) but a lower gas production (*p* < 0.05) than Futura 75. These results are clearly illustrated by the gas production over time shown in Figure 2, where the 8 mm and Futura 75 samples reached higher levels than the 6 mm and Uso 31 samples, and where Futura 75 tended to be faster than Uso 31, reaching maximum fermentation rate in less time. Irrespective of the nozzle size and the variety of hemp, the fermentation rate reached its peak after 3–6 h of incubation (Figure 3).

Table 5 shows the end products after 48 h of incubation. No effect of nozzle size was observed, whereas Uso 31 showed higher pH value and production of total VFAs (*p* < 0.01) because of increased production of acetate, propionate, branched chain fatty acids (*p* < 0.01), isovalerate and valerate (*p* < 0.05). The iterations observed for acetate, valerate, total VFA and branched chain fatty acids reflect the different trends of these molecules in respect to nozzle diameter and variety.

**Table 5:** *In vitro* fermentation end-products (mmol/l, if not otherwise stated).

Item	Nozzle diameter		Variety		SEM	Significance^1^		
	8 mm	6 mm	Futura 75	Uso 31		Diameter	Variety	Interaction
pH	7.00	7.01	6.97	7.03	0.001	NS	***	NS
Acetate	29.51	29.24	27.32	31.43	5.15	NS	***	*
Propionate	8.69	8.22	7.85	9.07	0.63	NS	***	NS
Iso-Butyrate	1.40	1.29	1.19	1.49	0.02	NS	***	NS
Butyrate	3.99	4.13	3.28	4.83	0.19	NS	***	NS
Iso-Valerate	2.39	2.36	2.26	2.50	0.04	NS	*	NS
Valerate	1.77	1.88	1.72	1.92	0.06	NS	*	**
VFA	47.76	47.12	43.64	51.23	10.26	NS	***	*
BCFA, % VFA	7.93	7.80	7.94	7.79	0.0028	NS	NS	**

^1^*p ≤ 0.05; **p ≤ 0.01; ***p ≤ 0.001; NS (not significant) indicates *p* > 0.10. SEM, standard error of means.

VFA, Volatile Fatty Acids; BCFA, Branched-Chain Fatty Acids.

## 4 Discussion

Due to the still small market for fiber, hemp cultivation in Italy focuses on seed production, although yields are often lower than in cooler environments, especially for early flowering varieties such as Uso 31 (19, 34). Observed seed yields are comparable to those reported for similar varieties grown under Mediterranean growing conditions (35–38). However, the levels of protein and fat, the most valuable macronutrients in hemp, were at the lower end of the range reported (7, 39, 40), suggesting a high incidence of empty seeds. On the other hand, the nutritional composition of the cakes was comparable to that of other medium to high protein feeds such as sunflower cake (40, 41) and as such may contribute to meeting the recommended dietary allowances for maintenance and growth of ruminants (42) and to supporting milk production in small ruminants and buffaloes during early lactation (42, 43). During mechanical extraction of oilseeds, the initial set temperature in the press chamber tends to increase due to friction. This can lead to an increase in the temperature of the oil and cake, affecting their chemical and physical properties (44, 45). Overall, the functional parameters of the press showed that the cooling system we implemented made it possible to limit the increase in extraction temperature, even when operating at a low screw speed (22 rpm). The small but significant increase in temperature, both in the compression zone and in the oil, observed when using the 6 mm nozzle compared to the 8 mm one, confirms previous research results highlighting the relationship between bushing diameter and extraction temperature (15, 46). Moreover, the temperature values of the extracted oils (T2) were found to be lower than those reported in other studies on tobacco seeds (47) and hemp seeds (22), both extracted with identical parameters as our mechanical press but without cooling equipment, further confirming the effectiveness of the system applied to the press barrel. This finding is also in line with the conclusions of Rabadán et al. (46), where heat transfer was identified as a significant factor contributing to temperature increases in oils and cakes extracted by mechanical methods. The lack of influence of hemp variety or bushing diameter on extraction times or operational capabilities is consistent with the constant extraction speed (ω: 22 rpm). It is worth noting that both extraction times and operational capabilities are closely related to the variation of the screw speed, as elucidated in previous studies (21, 22). Finally, the absence of significant differences between the two nozzle sizes in terms of extraction time and machine operating capacity, and the fact that any additional costs due to energy consumption are marginal compared to the benefits of the increased quantity of hemp oil extracted, indicate that the use of smaller nozzles during the extraction process is an economically viable option.

The differences in oil yield and cake composition by using 6- or 8-mm nozzles were expected. Indeed, the higher pressure exerted by the 6 mm nozzle allows a higher oil extraction and thus a lower percentage of fat in the corresponding residual cake, which in turn influence the percentages of the other components. Furthermore, given the high retail price of hempseed oil, the use of the 6 mm diameter appears to be the most worthwhile option.

The better oil yield observed for Uso 31 is not easy to explain given the fairly uniform fat content of the untreated seeds of the two varieties and the lack of influence of variety on functional parameters of the press. It could be hypothesized that a different organization of seed tegument structures and/or fat vesicles in the endosperm may offer different resistance to applied pressure, but further research is needed to confirm and explain the effect of hemp variety on oil extraction efficiency. As a result of the higher oil yield, the Uso 31 cakes had a lower fat content, corresponding to a higher fiber percentage, and a lower protein and higher ash content, in line with the composition of the unpressed seed. Irrespective of nozzle diameter and variety, the cake had a lower protein and higher structural carbohydrate content than reported in the literature (5, 7, 47), reflecting the characteristics found for unprocessed hempseeds.

For the *in vitro* study, the differences observed between the 6 and 8 mm cakes in organic matter digestibility could be related to the higher fiber content, in particular lignin, of the 8 mm cake, which could have reduced the rate, and the speed of the carbohydrate fermentation processes (48, 49). The slightly lower lignin content of the 8 mm cakes may also have played a role in the higher OVMD. Lignin is a complex aromatic polymer consisting of phenylpropane units that increases in the cell wall as the plant matures (50). Lignin plays a central role in plant growth and development by improving water conduction through xylem vessels, increasing the strength of fibrous tissues, and limiting the spread of pathogens in plant tissues, but it also reduces the degradation of structural polysaccharides by hydrolytic enzymes, thereby limiting nutrient availability (51). There were significant differences between the cakes of the different varieties, with Futura 75 showing lower OMD compared to Uso 31, probably due to its higher ADL (+2.8%) content. In this respect, all samples showed a low percentage of organic matter degradability (OMD), probably related to the high presence of lignin (>10% DM). However, the active molecules present in hemp could cause an overestimation of lignin, as suggested by Marles et al. (52) for substrates rich in condensed tannins. The *in vitro* concentration of the end products was not influenced by the nozzle diameter, whereas the Futura 75 variety showed a lower concentration for all VFA compared to Uso 31, most likely related to the lower degradability of organic matter, which reduces VFA absorption. In ruminants, VFA production is mainly due to the digestion of carbohydrates (53). The high content of lignin in hempseed cake could reduce carbohydrate digestion and reduce VFA production. Several studies in the literature [see the reviews by Bailoni et al. (54, 55); Šťastník et al. (56); Rakita et al. (40)] indicated that hemp seed cake could be an alternative feed ingredient, a good source of protein and lipid with a balanced amino acid and fatty acid profile. However, conflicting results have been found on the productive performance of animals, depending on several factors such as the type and chemical composition of the cakes, the level of inclusion in the diet, the category of animal and the duration of the experiment (40). Furthermore, Karlsson and Martinsson (57) tested hemp seed cake in an *in-situ* experiment and reported low protein digestibility due to a high content of indigestible NDF (iNDF). Lipids not only influence the fatty acid profile of foods of animal origin, but also make it possible to increase the energy concentration of ruminant diets. On the other hand, it should be noted that an excessive apport of dietary lipids can reduce feed intake and severely disrupt rumen fermentation, leading to a reduced digestibility of non-lipid energy sources, thus nullifying the benefits of increased dietary energy density (58). Although limited in number, *in vivo* studies evaluating cold-pressed hempseed cake in ruminant diets suggest that it may serve as a safe dietary supplement for both growing and lactating animals, primarily as an alternative protein or PUFA-rich fat source (40, 54, 55). Feeding hempseed cake to lactating ewes increased milk yield and, in terms of quality, resulted in higher fat and PUFA content, a more favorable ratio of n-6 to n-3 fatty acids, and increased levels of alpha-tocopherol and antioxidants (59). Similar results were recently reported by Šalavardić et al. (60) in Alpine dairy goats fed 60 g/kg hempseed cake as a replacement for soybean meal. Karlsson et al. (61) suggest a hempseed cake dose of 143 g/kg concentrate for lactating cows. In growing ruminants, diets supplemented with hempseed cake were able to achieve similar average daily gains in young calves and lambs compared to diets based on soybean meal or barley (57, 62, 63).

## 5 Conclusion

The use of food industry residues for animal feed is a key factor in achieving circular farming systems and more sustainable livestock production, but the high compositional variability associated with both the raw material and the industrial process may prevent their widespread use. The effect of using different nozzle sizes on cold oil extraction efficiency and residual cake characteristics was investigated using two different varieties of hemp. The cooling system kept the increase in extraction temperature within an acceptable range, allowing the use of smaller nozzle sizes to increase oil yield while reducing potential temperature-related damage to the hemp oil. The unprocessed seeds were rather low in fat and protein and these characteristics were reflected in the composition of the remaining cakes. The use of different nozzle sizes had small, though significant, effects on the characteristics of the residual cakes, both in terms of chemical composition and *in vitro* ruminal kinetics, although further effects on fatty acid composition and nutraceutical biomolecule content should be investigated. Overall, the use of a 6 mm diameter in a temperature-controlled extraction process allows a higher yield of hemp oil without adversely affecting the residual cake as a source of dietary protein and fat for ruminants. Although differences in the composition of the starting hemp seeds are unavoidable, further investigations analyzing the effect of the nozzle on the fatty acid profile of the cakes, together with *in vivo* feeding trials in both ruminant and monogastric species, will be of interest to fully validate the results of the present study.

## Data availability statement

The raw data supporting the conclusions of this article will be made available by the authors, without undue reservation.

## Ethics statement

The animal study was approved by Ethical Animal Care and Use Committee of the University of Napoli Federico II (Prot. 2019/0013729 of 08/02/2019). The study was conducted in accordance with the local legislation and institutional requirements.

## Author contributions

MS: Conceptualization, Formal analysis, Investigation, Methodology, Writing – original draft, Mechanical oil extraction. AV: Conceptualization, Data curation, Formal analysis, Investigation, Methodology, Resources, Writing – original draft, In vitro fermentation study. SF: Funding acquisition, Resources, Supervision, Visualization, Writing – original draft. FM: Data curation, Formal analysis, Validation, Writing – original draft, Writing – review & editing. ADF: Funding acquisition, Project administration, Resources, Supervision, Visualization, Writing – original draft. FSa: Investigation, Resources, Writing – original draft. MP: Investigation, Writing – original draft. DdK: Investigation, Writing – original draft. FSe: Conceptualization, Formal analysis, Writing – original draft, Writing – review & editing.
